# 

**Published:** 1894-05

**Authors:** 


### CONTENTS FOR MAY, 1894.

ORIGINAL COMMUNICATIONS. PAGE.

The Physician's Personality and Its Influence. By J. C. THOMPSON, M. D. .................... 577
The Life and Work of Charcot. By WILLIAM C. KRAUSS, M. D. .................... 582

CLINICAL REPORT.

Clinical Memoranda from the Surgical Clinic at the Sisters of Charity Hospital. By HERMAN MYNTER, M. D. .................... 588

SOCIETY PROCEEDINGS.

Buffalo Academy of Medicine .................... 590

TRANSLATION.

Late Researches in Epilepsy .................... 597

SELECTIONS.

Functional Dyspepsia, So-called .................... 602
The Physiology of Conception .................... 605
The Treatment of Scarlet Fever .................... 608
Some Suggestions on Life Insurance .................... 609

EDITORIAL.

State Examination and Public Health .................... 613
The Fog Horn Nuisance .................... 615
National Conference of State Medical Examining and Licensing Boards .................... 616
Topics of the Month .................... 617
Personal .................... 624
Obituary .................... 624
Society Meetings .................... 625
College Notes .................... 627
Book Reviews .................... 628
Literary Notes .................... 639

